# Pathogenic characteristics of pulmonary infection in hospitalized patients with chronic heart failure and diagnostic value of sTREM-1, sCD163, and sTWEAK

**DOI:** 10.12669/pjms.38.3.4758

**Published:** 2022

**Authors:** Fang Zheng

**Affiliations:** 1 Fang Zheng Department of Cardiology, Baoding Huayi Hospital of Traditional Chinese Medicine, Baoding, Hebei 071000, China

**Keywords:** Chronic heart failure, Pulmonary infection, sTREM-1, sCD163, sTWEAK

## Abstract

**Objective:**

To investigate the pathogenic characteristics of pulmonary infection in hospitalized patients with chronic heart failure as well as the diagnostic value of soluble myeloid cell expression triggering receptor-1 (sTREM-1), soluble CD163 (sCD163) and soluble tumor necrosis factor-like weak inducing factor (sTWEAK).

**Methods:**

A total of 72 patients with pulmonary infection who were hospitalized with chronic heart failure from December 2017 to December 2019 in the Department of Cardiology of Hebei Baoding Huaying Hospital of Traditional Chinese Medicine, China, were selected as the infection group, seventy-two patients without pulmonary infection who were hospitalized with chronic heart failure were selected as the non-infection group, and 50 healthy subjects who underwent physical examination in the hospital during the same period were selected as the control group. The distribution characteristics of pathogens in the infection group were statistically analyzed. The levels of sTREM-1, S CD163 and STweak in serum of patients with different infection severity and different cardiac function grades were compared among the three groups. Receiver operating characteristic curve (ROC) was utilized to evaluate the predictive value of the three indicators for the adverse prognosis of patients in hospital.

**Results:**

A total of 76 strains of pathogens were cultured from two hospitalized patients with pulmonary infection of chronic heart failure, among which 43 strains (56.58%) were gram-negative bacteria, 29 strains (38.15%) were gram-positive bacteria, and four strains (5.26%) were fungi. The levels of sTREM-1 and sCD163 in the control group, non-infection group and infection group were gradually increased (*p<*0.05), while there was no difference in sTWEAK between the infection group and the non-infection group (*p*>0.05). In the infection group, the expression levels of sTREM-1 and sCD163 increased with the severity of infection, with statistically significant differences (*p<*0.05), while there was no statistically significant difference in the expression level of sTWEAK among different infection severity (*p*>0.05). The higher the cardiac function grade of patients in the infection group, the higher the levels of sTREM-1 and sCD163, and the lower the level of sTWEAK, with a statistical significance (*p*<0.05). ROC analysis results showed that the serum sTREM-1, sCD163, and sTWEAK levels for the poor prognosis of patients with CHF combined with lung infection had areas under the curve of 0.864, 0.870, and 0.822, respectively, and the 95% CI values were 0.787-0.941, 0.795-0.945 and 0.733-0.910, respectively, all *p*<0.001.

**Conclusions:**

Pulmonary infection in hospitalized patients with chronic heart failure is mainly caused by gram-negative bacteria. Detection of sTREM-1, sCD163, and sTWEAK levels is of certain value in judging the condition and prognosis, which is worthy of clinical promotion.

## INTRODUCTION

According to the survey of “Report on Cardiovascular Diseases in China (2017)”, the prevalence of cardiovascular diseases in China continues to rise, and deaths caused by cardiovascular diseases rank as the primary cause of death among urban and rural residents in China.^1^ Pulmonary infection, as one of the most common complications in patients with chronic heart failure (CHF), especially in elderly patients, is difficult to be diagnosed because the clinical symptoms are not obvious in the early stage. Investigating the characteristics of its pathogens is helpful for physicians to accurately select antimicrobial agents, improve the therapeutic effect and reduce the burden on patients.^2^,^3^ It has been reported in previous studies that some commonly used laboratory indicators, such as C-reactive protein (CRP) and procalcitonin (PCT), have been used in the clinical diagnosis and prognosis of pulmonary infection in patients with CHF. However, the clinical diagnosis and prognosis of pulmonary infection are not ideal owing to the problem of reasonable sensitivity but insufficient specificity of these indicators. It is, therefore, a trend of future research to combine multiple biomarkers for the diagnosis and prognosis of diseases.^4^,^5^

Soluble myeloid cells express trigger receptor-1 (sTREM-1) is a soluble subtype of myeloid cell triggering receptor-1, which is distributed in body fluids or blood and can mediate the inflammatory cascade. It is mainly applied in the diagnosis of sepsis, postoperative fracture infection and abdominal cavity infection.^6^,^7^ Soluble CD163 (sCD163), a soluble form of scavenger receptor family CD163, mainly mediates anti-inflammatory and antioxidant reactions.^8^ The expression of soluble tumor necrosis factor-like weak apoptosis-inducing factor (sTWEAK) is regulated by sCD163. sCD163/STWEAK has been found to reflect the severity of acute coronary syndrome and chronic heart failure.^9^,^10^ The expression changes of the three indicators and their clinical value in patients with CHF complicated with pulmonary infection remain unclear. In this study, the pathogenic characteristics of pulmonary infection in hospitalized patients with chronic heart failure and the diagnostic value of sTREM-1, sCD163 and STweak were investigated, aiming to provide valuable reference for clinical diagnosis and treatment of this disease. The results are reported as follows.

## METHODS

### Subjects

A total of 72 patients with pulmonary infection who were hospitalized with chronic heart failure from December 2017 to December 2019 in the Department of Cardiology, Huaying Hospital of Traditional Chinese Medicine in Baoding, Hebei Province were selected as the subjects and included in the infection group. Moreover, a total of 72 patients without pulmonary infection who were hospitalized with chronic heart failure were randomly selected as the non-infection group in a ratio of 1:1.

### Ethical Approval

The study was approved by the Institutional Ethics Committee of Baoding Huayi Hospital of Traditional Chinese Medicine on June 20, 2018 (No. (2017)03), and written informed consent was obtained from all participants.

### Inclusion criteria:

1. Patients diagnosed with CHF by sign examination and echocardiography, and meeting the corresponding criteria in the Chinese Guidelines for the Diagnosis and Treatment of Heart Failure 2018.^11^
2. Patients whose diagnosis of pulmonary infection meets the diagnostic criteria of nosocomial infection.^12^
3. Patients with New York cardiac function grade II to IV.
4. Patients with complete medical records.

### Exclusion criteria:

1. Patients complicated with infection of other sites.
2. Patients with acute myocardial infarction and acute myocarditis whose course of disease was < 1 month.
3. Patients transferred to hospital during treatment.
4. Patients with malignant tumors or infectious diseases.

Fifty healthy subjects who underwent physical examination in the same hospital were selected as the control group. The gender, age and primary disease of the three groups were statistically analyzed via electronic medical record survey.

Respiratory secretions were collected aseptically from suspected infected patients, and pathogenic bacteria were identified using SHERLOCK ® Microbial Identification System (MIDI, USA).

Patients were divided into NYHA grade II group, NYHA grade III group and NYHA grade IV group according to their cardiac function. Clinical Pulmonary Infection Score (CPIS) was used to grade the severity of pulmonary infection, including body temperature, white blood cell count, tracheal secretions, oxygenation, chest X-ray, and progress of lung infiltrating image, etc., with the highest score of 12, Mild: 6-7 points; Moderate: 8-9 points; Severe: 10-12 points. According to the results, patients in the infection group were divided into mild infection group, moderate infection group and severe infection group. Patients in the infection group were followed up after the diagnosis of infection, and the death rate within 60 days was statistically analyzed.

Two milliliter peripheral elbow venous blood from all patients was aseptically extracted and centrifuged to prepare as serum. Subsequently, enzyme-linked immuno sorbent assay (ELISA) was used to detect the levels of sTREM-1, sCD163 and sTWEA in serum. Three duplicate wells were designed for each sample. The kit was purchased from Shanghai Enzyme Linked Biology Co., Ltd. The enzyme plate instrument was Multiskan FC enzyme plate instrument (Theomofisher Company, USA).

### Statistical Analysis

SPSS 21.0 was used to analyze the collected experimental data. In the experimental data, the measurement data conforming to normal distribution was represented by`X±S. The independent sample t test was used for comparison between two groups, and the F test was used for comparison between multiple groups. Encounter data were represented as the number of cases or rate. *χ*^2^ test was used for comparison between the two groups, and death on the 60d was taken as the adverse prognosis outcome. Receiver operating characteristic (ROC) was used to evaluate the prognostic value of the three indicators. P<0.05 indicates a statistically significant difference.

## RESULTS

There was no statistical significance in gender, age, primary disease and other general information among the three groups (*p*>0.0.5), as shown in Table-I.A total of 76 strains of pathogens were cultured from 2 hospitalized patients with pulmonary infection of chronic heart failure, among which 43 strains (56.58%) were gram-negative bacteria, 22 strains (38.15%) were gram-positive bacteria, and 4 strains (5.26%) were fungi Table-II.

**Table-I:** Comparison of general information between the three groups.

Item		Infection group (n=72)	non-infection group (n=72)	Control group (n=50)	Statistics	p-value
Gender	Male	43	38	27	0.776	0.679
	Female	29	34	23		
Age (years old)		60.67±5.16	61.02±5.22	59.37±5.08	1.598	0.205
Primary disease	Ischemic cardiomyopathy	41	45	-	0.333	0.999
	Hypertensive heart disease	16	14	-		
	Dilated cardiomyopathy	10	7	-		
	Valvular heart disease	5	6	-		

**Table-II:** Infection pathogen distribution.

Pathogen	Number of stains (n=76)	Constituent ratio
Gram-negative bacteria	43	56.58
Pseudomonas aeruginosa	17	22.37
Klebsiella pneumoniae	13	17.11
Acinetobacter baumannii	6	7.89
Haemophilus influenzae	4	5.26
E.coli	3	3.95
Gram-positive bacteria	29	38.16
Staphylococcus aureus	12	15.79
Streptococcus pneumoniae	8	10.53
Coagulase Staphylococcus	5	6.58
Dung enterococcus	4	5.26
Fungi	4	5.26
Candida albicans	3	3.95
Candida parapsilosis	1	1.32

There were statistically significant differences in sTREM-1, sCD163 and sTEAK levels among the three groups (*p*<0.05). The levels of sTREM-1 and sCD163 in the infection group were higher than those in the non-infection group and the control group, with statistically significant differences (*p*<0.05). The levels of sTREM-1 and sCD163 in the non-infection group were higher than those in the control group, with statistically significant differences (*p*<0.05). No statistically significant difference was observed in the sTWEAK level between the infection group and the non-infection groups (*p*>0.05), while the sTWEAK level between the infection and the non-infection group was lower than that of the control group (*p*<0.05). Table-III.

**Table-III:** Comparison of the levels of sTREM-1, CD163 and sTWEAK among the three groups (*X*¯±S).

Indicators	Infection group (n=72)	non-infection group (n=72)	Control group (n=50)	Statistics	p-value
sTREM-1 (pg/mL)	64.28±18.27	41.18±15.08	20.91±10.24	120.561	<0.001
sCD163 (ng/mL)	134.24±35.81	93.11±28.94	61.57±18.39	92.41	<0.001
sTWEAK (ng/mL)	234.15±53.64	251.34±60.24	533.27±101.33	311.044	<0.001

One-way ANOVA results showed that in the infection group, the more severe the infection was, the higher the expression levels of sTREM-1 and sCD163 were, with a statistically significant difference (*p*<0.05). There was no statistically significant difference in the expression level of sTWEAK among different infection severity (*p*>0.05 Table-IV.

**Table-IV:** Comparison of sTREM-1, CD163 and sTWEAK levels of different infection severity(*X*¯±S).

Indicators	Mild infection group (n=31)	Moderate infection group (n=26)	Severe infection group (n=15)	Statistics	p- value
sTREM-1 (pg/mL)	43.49±11.62	60.19±15.84	74.19±16.92	27.724	<0.001
sCD163 (ng/mL)	112.53±24.15	134.27±27.52	157.52±29.46	15.120	<0.001
sTWEAK (ng/mL)	223.41±40.32	241.08±39.67	219.34±42.31	1.878	0.161

One-way ANOVA results showed that the higher the cardiac function grade of patients in the infection group, the higher the levels of sTREM-1 and sCD163, and the lower the level of sTWEAK, with a statistical significance (*p*<0.05). Table-V.

**Table-V:** Comparison of sTREM-1, sCD163 and sTWEAK levels of different cardiac function grades (*X*¯±S).

Indicators	NYHA Grade II group (n=34)	NYHA Grade III group (n=24)	NYHA Grade IV group (n=44)	Statistics	p-value
sTREM-1 (pg/mL)	41.66±12.37	59.88±14.28	72.57±17.34	26.708	<0.001
sCD163 (ng/mL)	110.37±20.55	131.60±24.82	160.34±30.11	21.706	<0.001
sTWEAK (ng/mL)	296.35±39.67	255.45±42.15	212.64±32.67	23.927	<0.001

**Fig.1:**
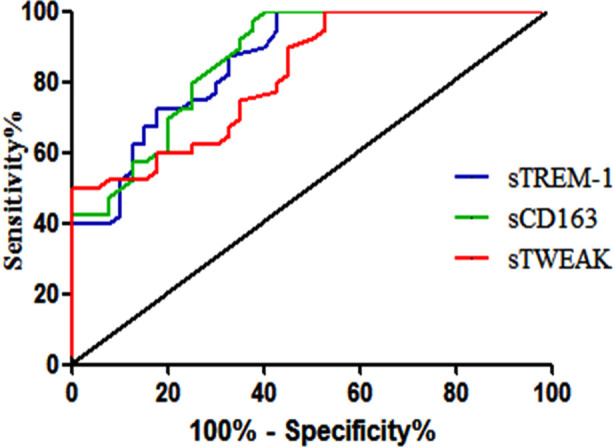
Predicted price of sTREM-1, sCD163, and sTWEAK levels for patients with poor prognosis.

ROC analysis results showed that the serum sTREM-1, sCD163, and sTWEAK levels for the poor prognosis of patients with CHF combined with lung infection had areas under the curve of 0.864, 0.870, and 0.822, respectively, and the 95% CI values were 0.787-0.941, 0.795-0.945 and 0.733-0.910, respectively, with statistical significance (*p*<0.001). Fig.1.

## DISCUSSION

Heart failure refers to the terminal stage of various heart diseases, which are clinically manifested by pulmonary congestion (dyspnea, lung rales), systemic congestion (peripheral edema), and reduced exercise tolerance. The prevalence rate among adults in China is as high as 0.9%, which accounts for 1% - 2% in developed countries, causing great harm.^13^,^14^ Pulmonary infection and heart failure affect each other and are causal to each other. Specifically, patients with heart failure have poor immune function, which can affect pulmonary circulation and increase the risk of lung infections. Pulmonary infections, in turn, can induce or aggravate existing heart disease, leading to complications such as ischemic heart disease, heart failure, and arrhythmias. At the same time, the inflammatory state of the lungs activates the systemic inflammatory response, with vascular tension changes, pulmonary circulation resistance significantly increased, the body oxygen consumption increased, the heart burden increased, thereby aggravating the heart failure. Pulmonary infection can also affect the body’s coagulation function, causing acute coronary syndrome and myocardial infarction.^15^,^16^ In this study, the pathogen distribution characteristics of this disease are first investigated, and the results show that the gram-negative bacteria accounted for the largest proportion, and the pathogens with a high detection rate include: Pseudomonas aeruginosa, Klebsiella pneumoniae, Staphylococcus aureus, etc. All these pathogens are the common causes of nosocomial infection, and have close bearing on medical equipment, operation of medical staff and the health awareness of patients and their families. Therefore, the prevention of hospital sensation should be strengthened in the work to minimize the occurrence of such nosocomial infection, so as to improve the prognosis of patients to the maximum extent and reduce adverse reactions.^17^,^18^

In the diagnosis of CHF complicated with pulmonary infection, patients with heart failure will have changes in laboratory indicators such as fever and leukocytosis, which overlap with the manifestations of pulmonary infection.^19^ Therefore, there are certain difficulties in diagnosis. It is still not clear about the release mechanism of sTREM-1, but studies have shown that sRM-1 can promote the secretion and release of pro-inflammatory factors activated by target cells through transduction of molecular death-related protein 12, and synergistic amplification of inflammatory response with Toll-like receptors, which has been reported to have clinical value in the diagnosis and prognosis of a variety of infectious diseases.^20^ In patients with sepsis, serum sTREM-1 level has been found to be closely related to cardiac dysfunction and poor prognosis in patients with sepsis.^20^ In patients with acute myocardial infarction, sTREM-1 levels are significantly associated with all-cause mortality and adverse cardiac events.^21^ Macrophage infiltration plays a key role in the process of chronic myocardial inflammation in CHF patients, and sCD163 is a specific marker of macrophage activation, which is associated with a variety of infectious diseases and cardiovascular diseases.^22^ The expression of sCD163 can influence the concentration of sTWEAK. In the process of sCD163-positive phagocytes or sCD163-mediated inflammation, sCD163 can reduce the blood sTWEAK concentration by means of isolation.^23^ Therefore, sTWEAK/sCD163 is considered as one of the potential biomarkers for multiple adverse cardiovascular events.^24^ The expression of sTWEAK varies in different tissues and organs, with high expression in the heart, lung, brain, intestinal tract, etc., and low expression in the kidney and liver. In the cardiovascular system, myocardial cells, endothelial cells and smooth muscle cells all express Tweak or its ligand FN14 mRNA or mRNA synthesis products.^25^,^26^ In this study, the levels of sTREM-1, sCD163 and sTWEAK with different infection severity and cardiac function grades in the infection group, the non-infection group and the control group are investigated, and the results show that all three indicators could reflect the cardiac function grade of patients with CHF. sTREM-1 and sCD163 can distinguish infection and severity of infection, while sTWEAK decrease in the infection group and cannot reflect the severity of infection. To analyze the causes, sTREM-1, sCD163, and sTWEAK are all expressed in the heart or myocardial tissue, so they can reflect the cardiac functional state of CHF. However, in terms of reflecting infection, sTREM-1 and sCD163 are directly or indirectly involved in systemic infection and inflammatory response, while sTWEAK is not involved in this process. In the infection group, the decrease of sTWEAK level may also be caused by the further impairment of cardiac function caused by infection. In other words, sTREM-1 and sCD163 are not only involved in the process of pathological changes of the heart, but also closely related to the process of inflammation caused by infection.^27^,^28^ Finally, in this study, the predictive value of the three indicators for the prognosis of patients with CHF complicated with pulmonary infection is evaluated, and the results show that the three indicators all have favorable predictive value for the poor prognosis of patients, among which the area of sCD163 AUC is the largest.

### Limitations of the study

However, the diagnostic value of the three indicators for different pathogen infections has not been evaluated due to the limitation of the number of cases, and further studies are needed to make the three indicators widely applied in clinical practice.

## CONCLUSIONS

The results of this study show that the lung infections of patients with chronic heart failure in hospital are mainly gram-negative bacteria, and the detection of sTREM-1, s-CD163 and sTWEAK levels is of certain value in judging the condition and prognosis.

## References

[1] Wei JT, Liang B, Yang HY, Bian YF, Yang ZM (2019). Analysis of Clinical Charactistics of 739 Hospitalized Patients with Chronic Heart Failure Admitted to a Single Center from 2015 to 2018. J Shanxi Med University.

[2] Santema BT, Ouwerkerk W, Tromp J, Sama IE, Ravera A, Regitz-Zagrosek V (2019). Identifying optimal doses of heart failure medications in men compared with women:a prospective, observational, cohort study. Lancet.

[3] Fowler AA 3rd, Truwit JD, Hite RD, Morris PE, DeWilde C, Priday A (2019). Effect of Vitamin C Infusion on Organ Failure and Biomarkers of Inflammation and Vascular Injury in Patients With Sepsis and Severe Acute Respiratory Failure:The CITRIS-ALI Randomized Clinical Trial [published correction appears in JAMA. 2020;323(4):379. JAMA.

[4] Freiberg MS, Chang CH, Skanderson M, Patterson OV, DuVall SL, Brandt CA (2017). Association Between HIV Infection and the Risk of Heart Failure with Reduced Ejection Fraction and Preserved Ejection Fraction in the Antiretroviral Therapy Era:Results from the Veterans Aging Cohort Study. JAMA Cardiol.

[5] Jia Q, Li H, Zhou H, Zhang X, Zhang A, Xie Y (2019). Role and Effective Therapeutic Target of Gut Microbiota in Heart Failure. Cardiovasc Ther.

[6] Cao C, Gu J, Zhang J (2017). Soluble triggering receptor expressed on myeloid cell-1 (sTREM-1):a potential biomarker for the diagnosis of infectious diseases. Front Med.

[7] Michel CS, Teschner D, Wagner EM, Theobald M, Radsak MP (2017). Diagnostic value of sTREM-1, IL-8, PCT, and CRP in febrile neutropenia after autologous stem cell transplantation. Ann Hematol.

[8] Mascia C, Pozzetto I, Kertusha B, Marocco R, Del Borgo C, Tieghi T (2018). Persistent high plasma levels of sCD163 and sCD14 in adult patients with measles virus infection. PLoS One.

[9] Dirajlal-Fargo S, Sattar A, Kulkarni M, Funderburg N, McComsey GA (2017). Soluble TWEAK may predict carotid atherosclerosis in treated HIV infection. HIV Clin Trials.

[10] Acharya AB, Chandrashekar A, Acharya S, Shettar L, Thakur S (2019). Serum sTWEAK levels in chronic periodontitis and type 2 diabetes mellitus. Diabetes Metab Syndr.

[11] Heart Failure Group of Cardiovascular Branch of Chinese Medical Association, Heart Failure Professional Committee of Chinese Medical Doctor Association, Editorial Board of Chinese Journal of Cardiovascular Diseases. Chinese Heart Failure Diagnosis and Treatment Guidelines 2018 (2018). Chi J Heart Failure Cardiomyopathy (Chinese and English).

[12] Ministry of Health, People's Republic of China. Diagnostic Criteria of Nosocomial Infection (Trial) (2001). Chin J Med.

[13] Porcel JM (2018). Biomarkers in the diagnosis of pleural diseases:a 2018 update. Ther Adv Respir Dis.

[14] Higuchi ML, Kawakami JT, Ikegami RN, Reis MM, Pereira JJ, Ianni BM (2018). Archaea Symbiont of T. cruzi Infection May Explain Heart Failure in Chagas Disease. Front Cell Infect Microbiol.

[15] Xanthopoulos A, Starling RC, Kitai T, Triposkiadis F (2019). Heart Failure and Liver Disease:Cardiohepatic Interactions. JACC Heart Fail.

[16] Savvoulidis P, Butler J, Kalogeropoulos A (2019). Cardiomyopathy and Heart Failure in Patients with HIV Infection. Can J Cardiol.

[17] Cardoso JN, Del Carlo CH, Oliveira Junior MT, Ochiai ME, Kalil Filho R, Barretto ACP (2018). Infection in Patients with Decompensated Heart Failure:In-Hospital Mortality and Outcome. Arq Bras Cardiol.

[18] Platz E, Jhund PS, Claggett BL, Pfeffer MA, Swedberg K, Granger CB (2018). Prevalence and prognostic importance of precipitating factors leading to heart failure hospitalization:recurrent hospitalizations and mortality. Eur J Heart Fail.

[19] Liu ZP, Zhang Y, Bian H, He XR, Zhou YJ, Wang LJ (2016). Clinical application of rapid B-line score with lung ultrasonography in differentiating between pulmonary infection and pulmonary infection with acute left ventricular heart failure. Am J Emerg Med.

[20] Li Z, Zhang E, Hu Y, Liu Y, Chen B (2016). High Serum sTREM-1 Correlates with Myocardial Dysfunction and Predicts Prognosis in Septic Patients. Am J Med Sci.

[21] Wang YK, Tang JN, Shen YL, Hu B, Zhang CY, Li MH (2018). Prognostic Utility of Soluble TREM-1 in Predicting Mortality and Cardiovascular Events in Patients With Acute Myocardial Infarction. J Am Heart Assoc.

[22] Liu Q, Ou Q, Chen H, Gao Y, Liu Y, Xu Y (2019). Differential expression and predictive value of monocyte scavenger receptor CD163 in populations with different tuberculosis infection statuses. BMC Infect Dis.

[23] Silva RL, Santos MB, Almeida PL, Barros TS, Magalhaes L, Cazzaniga RA (2017). sCD163 levels as a biomarker of disease severity in leprosy and visceral leishmaniasis. PLoS Negl Trop Dis.

[24] Suzuki Y, Enomoto Y, Yokomura K, Hozumi H, Kono M, Karayama M (2016). Soluble hemoglobin scavenger receptor CD163 (sCD163) predicts mortality of community-acquired pneumonia. J Infect.

[25] Fan WC, Huang CC, Yang YY, Lin A, Lee KC, Hsieh YC (2017). Serum pentraxin-3 and tumor necrosis factor-like weak inducer of apoptosis (TWEAK) predict severity of infections in acute decompensated cirrhotic patients. J Microbiol Immunol Infect.

[26] Asil M, Dertli R (2016). Serum soluble TWEAK levels are decreased in treatment naive noncirrhotic chronic hepatitis B patients. Medicine (Baltimore).

[27] Sapa A, Rak A, Wybieralska M, Machon J, Krzywonos-Zawadzka A, Zawadzki K (2017). Diagnostic usefulness of sCD163, procalcitonin and neopterin for sepsis risk assessment in critically ill patients. Adv Clin Exp Med.

[28] Ptaszynska-Kopczynska K, Marcinkiewicz-Siemion M, Lisowska A, Waszkiewicz E, Witkowski M, Jasiewicz M (2016). Alterations of soluble TWEAK and CD163 concentrations in patients with chronic heart failure. Cytokine.

